# Supplementary material for: Episodes of fast crystal growth in pegmatites
Source: Nat Commun. 2020 Oct 5;11:4986. doi: 10.1038/s41467-020-18806-w (PMC7536386; doi:10.1038/s41467-020-18806-w)
Supplement: Supplementary file 1 — Supplementary Information [file 41467_2020_18806_MOESM1_ESM.pdf]

Supplementary material to:

Episodes of fast crystal growth in pegmatites

Phelps et al.

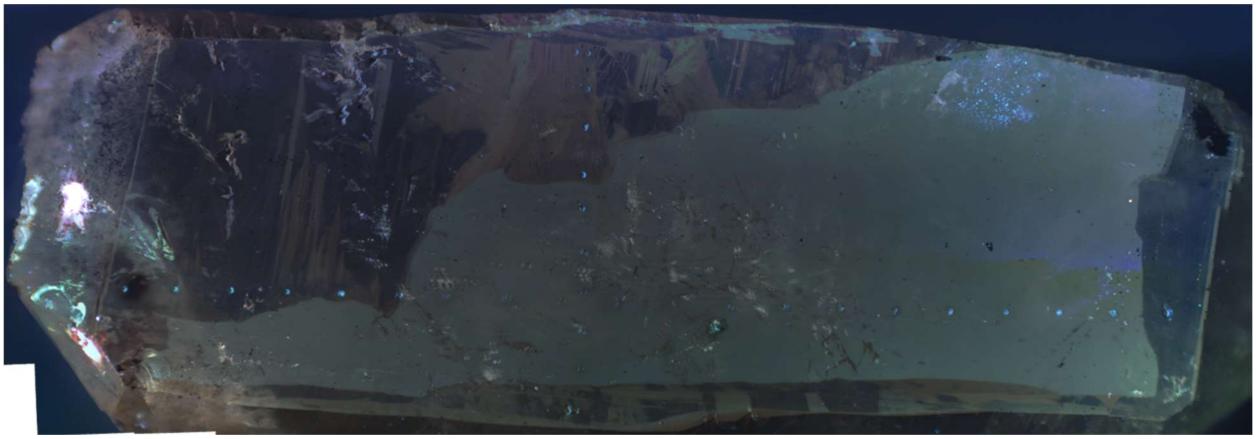

Supplementary Figure 1. **CL image of a quartz crystal from the Stewart pegmatite (STWC).**

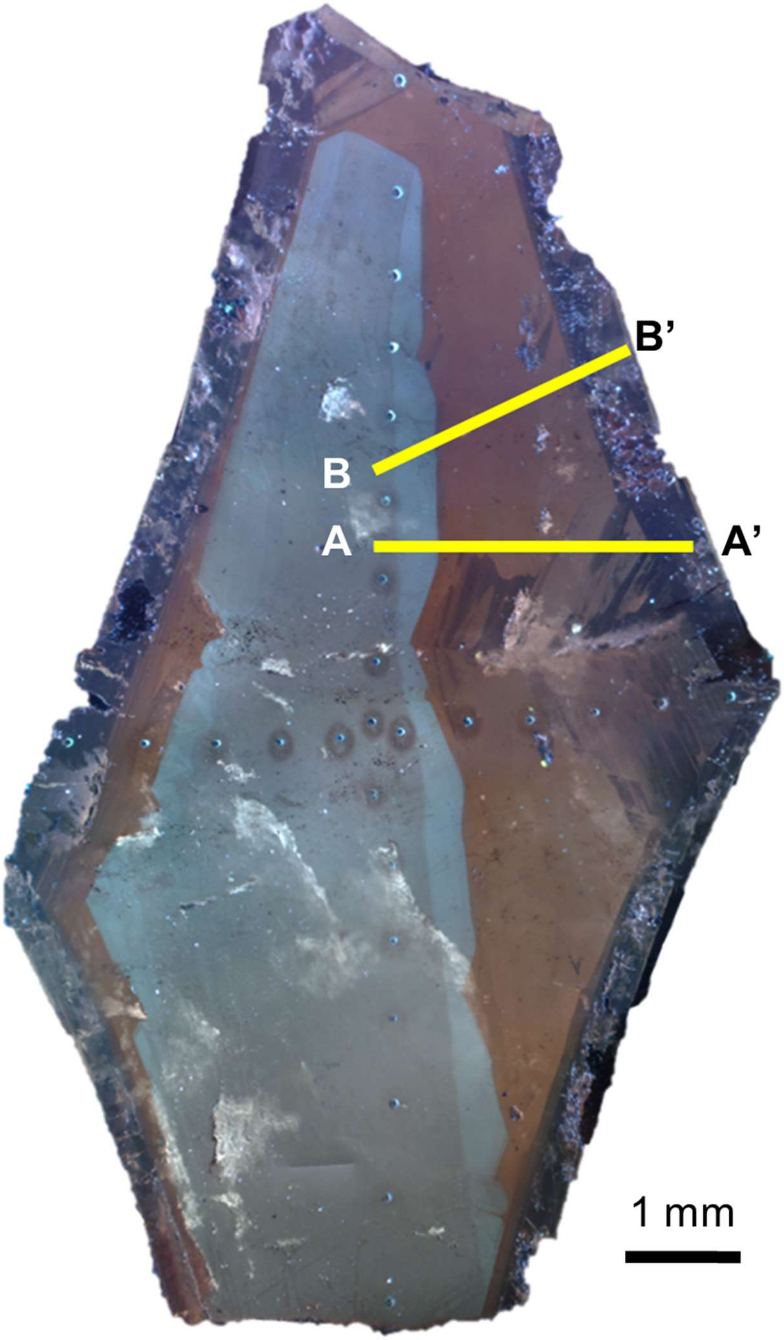

Supplementary Figure 2. **CL image of a quartz crystal from the Stewart pegmatite (STWF).**

This is the same as Figure 2a with the added transect B-B'. The data for this transect follows.

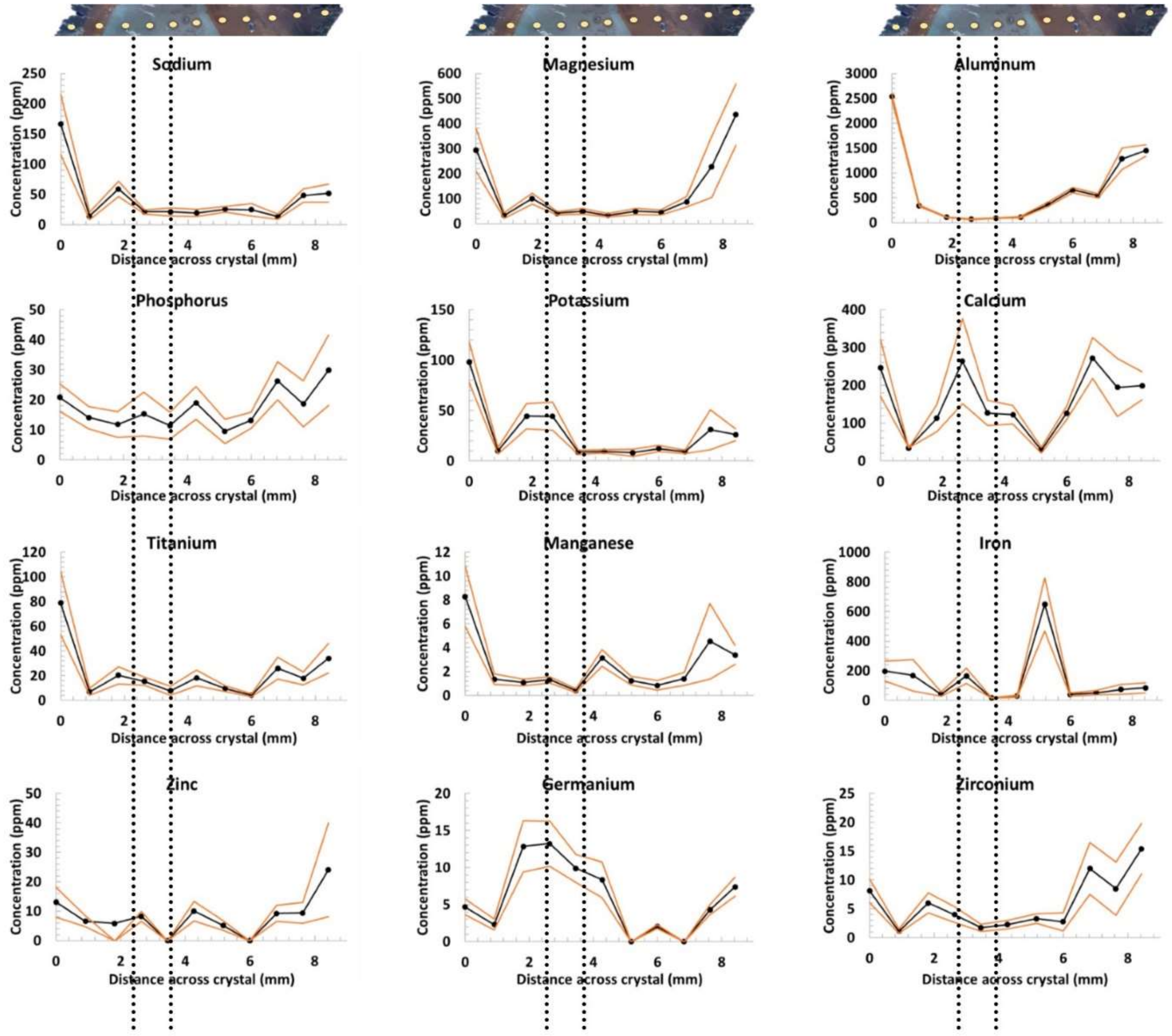

Supplementary Figure 3. **Point analyses for STWF.** 55µm spot size, 16 J/m^2 fluence. Black lines and points are data. Orange lines gives 3σ standard deviation for each point. CL images of the points are over top to indicate where in the crystal the analysis was done. Refer to Fig. 2a for larger image. Dashed lines demarcate the approximate center of the crystal.

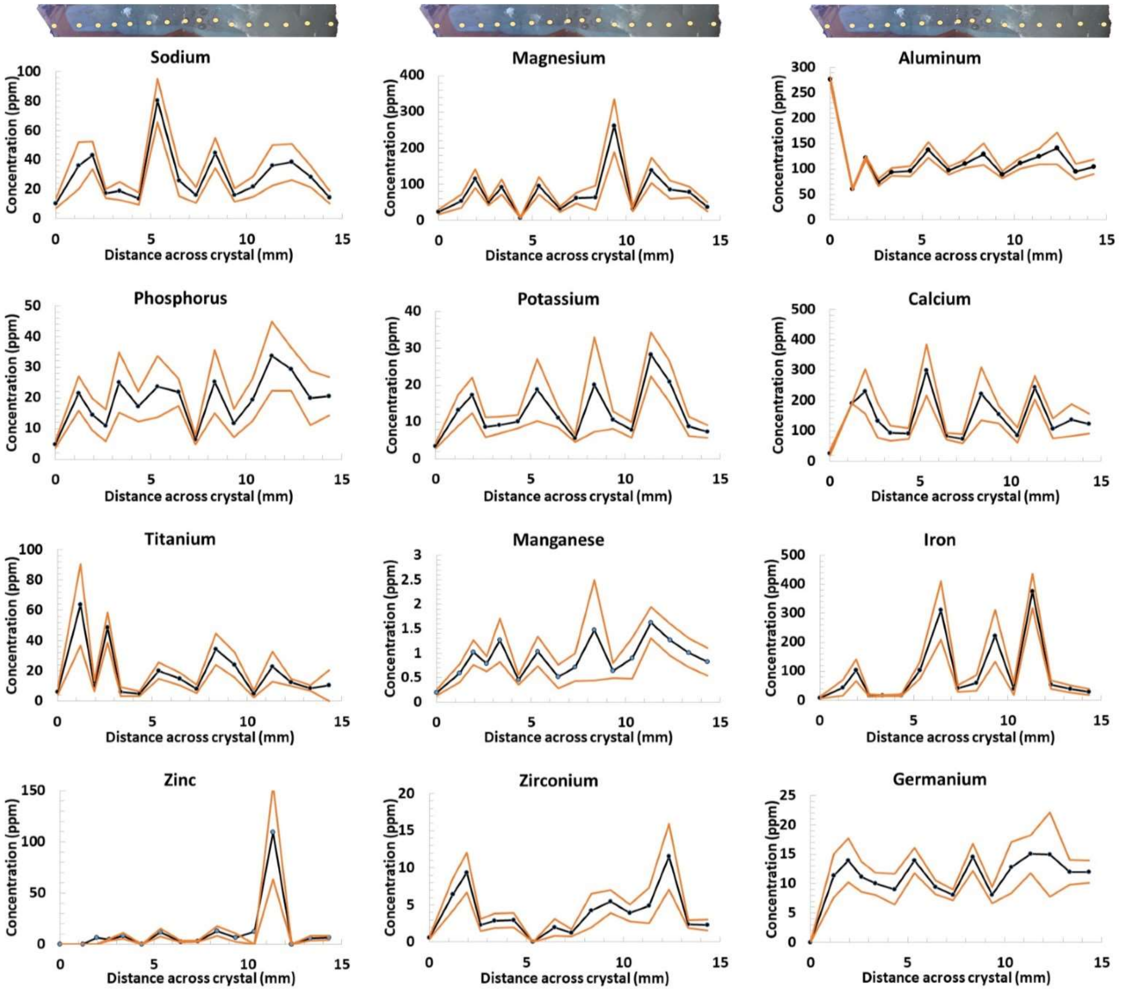

Supplementary Figure 4. **Continued point analyses for STWF along a different transect.**

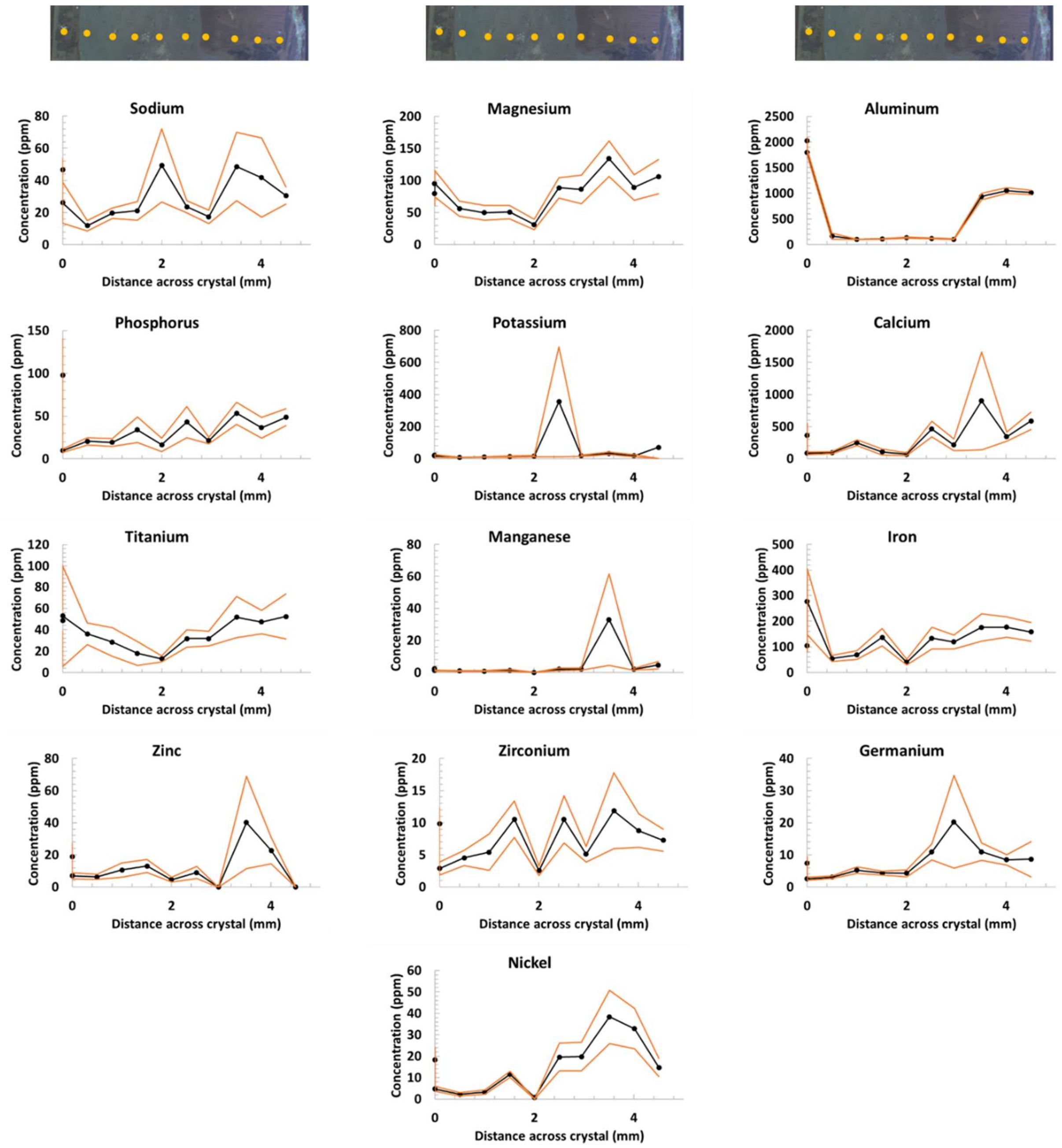

Supplementary Figure 5**. Point analyses for STWC.** Same analytical conditions as STWF.

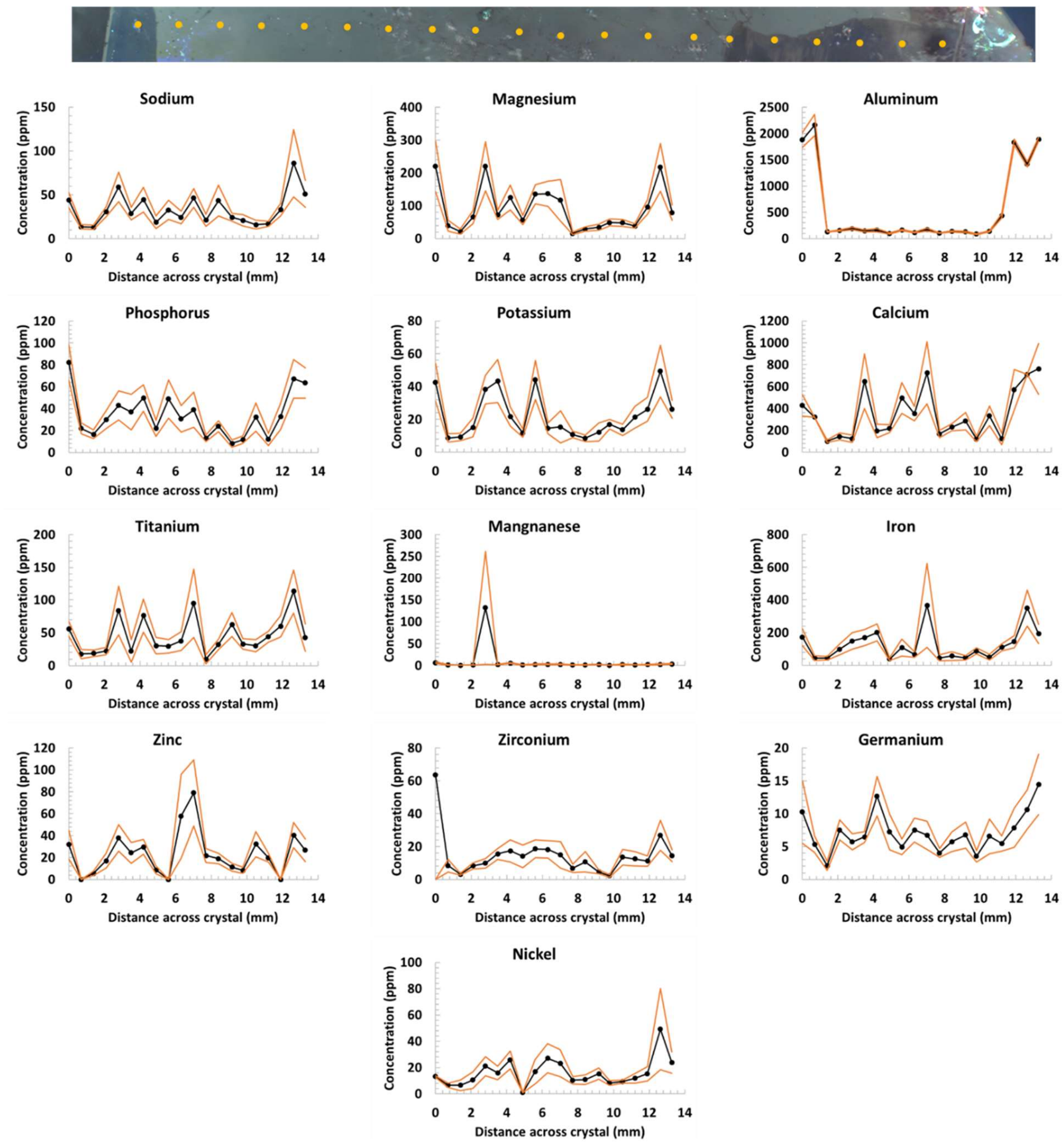

Supplementary Figure 6. **Continued point analyses for STWC along a different transect.**

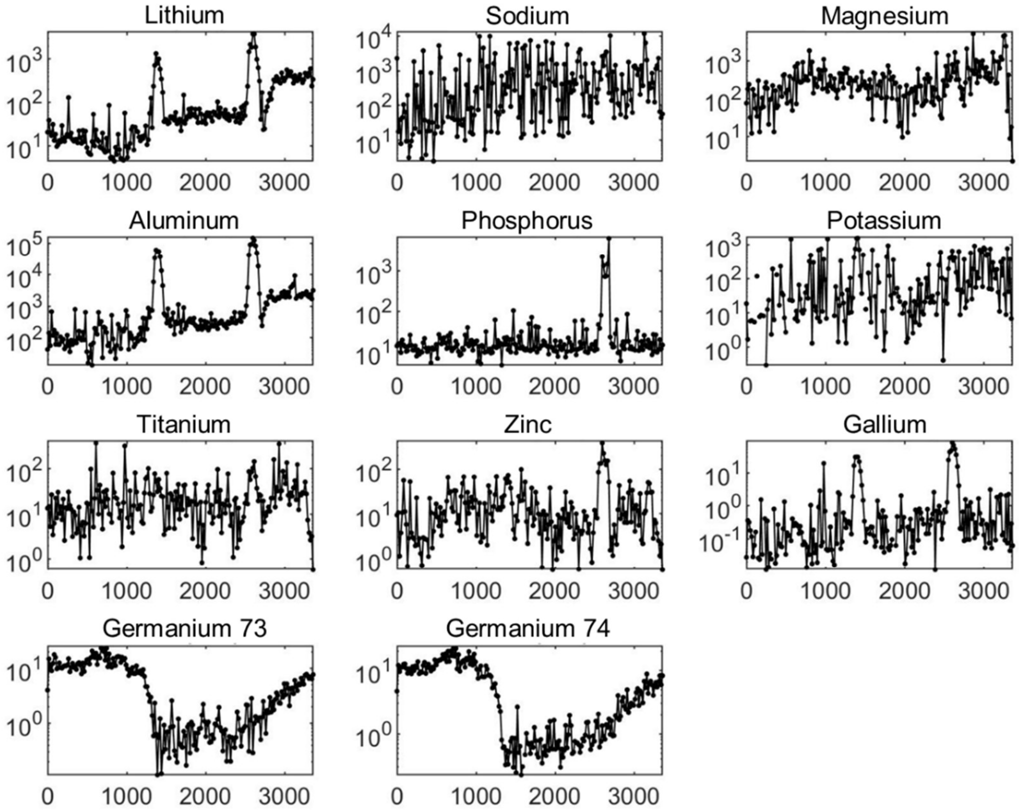

Supplementary Figure 7. **B-B' transect of Supp. Figure 2.** Y-axis is concentration in ppm, x-axis is distance in microns. Each plot shows the individual isotope measured with a black line representing the 3 point running average. Same conditions as A-A'.

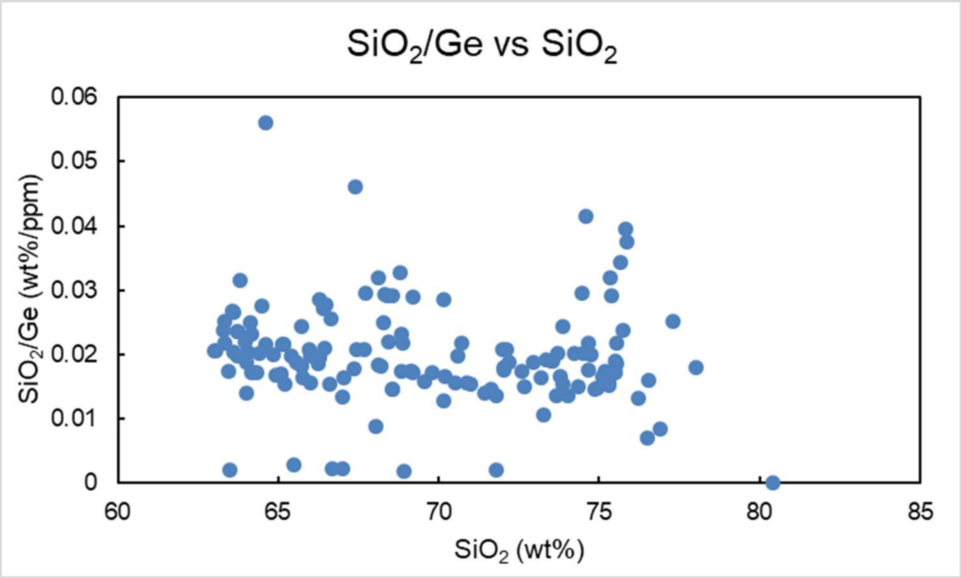

Supplementary Figure 8. **Global compilation of Ge and SiO_2 data.** SiO_2 (wt%) divided by Ge (ppm) plotted against SiO_2 (wt%). The slope is near zero, indicating Ge and SiO_2 behave similarly in granitic systems. All data was taken from EarthChem[1–12]. Data was chosen to ensure only high silica rocks were included. Converting wt% to ppm or vice versa is not necessary since we are only concerned with the slope.

Supplementary note 1:

<u>Accounting for laser smearing</u>

Measurements made with LA-ICPMS corresponds to an infinitesimal point at the center of the laser spot, yet the laser spot has a finite size and volume. This measurement is an average of the ablated volume. When measuring a transect, the laser interacts with portions of the sample that have already been measured as well as portions that will be measured in the future. These concentrations go into the averaging, smearing the concentration value at the point. For sharp

concentration changes, this can be a significant effect.

Model concentration profiles can be smeared based on this averaging and compared to measurements. Assuming concentration homogeneity with depth (only lateral concentration gradients), we can compute weighted averages by the area ablated. First, we discretize the spot into segments of a certain width, and then calculate the area of each segment. The segment width should correspond to the distance between data points. The weighting factor for each segment is then the ratio of the area of the segment to the total area of the spot. In mathematical terms: $w = \frac{A_{segment}}{A_{spot}}; A_{spot} = \pi\, r^2$, where w is the weighting factor and r is half of the spot size. Each segment's area is calculated moving from the outsize inward (see Supplementary Figure 9). The area of a segment is the area of a sector of the circle that includes the segment minus the area of the triangle that defines the inner portion of the sector and minus any previous segments.

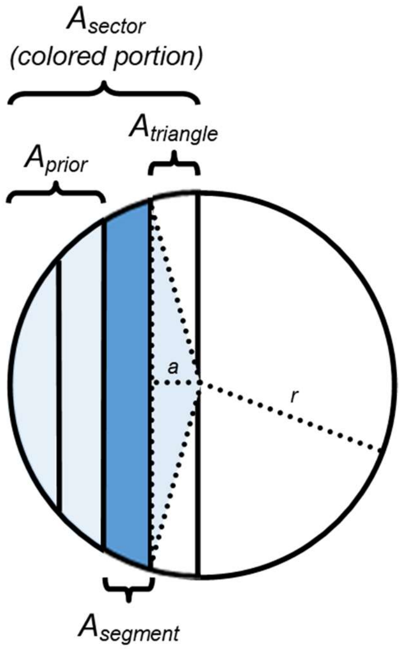

Supplementary Figure 9. **Schematic of how to calculate laser smearing.**

$$A_{segment} = A_{sector} - A_{triangle} - \sum A_{prior} \qquad Supplementary\ Eq. 1$$

$$A_{sector} = \frac{1}{2}r^2\theta;\ \theta = 2\arccos\left(\frac{a}{r}\right);\ A_{triangle} = a\sqrt{r^2 - a^2} \qquad Supplementary\ Eq. 2$$

where a is the radial distance to the inner edge of the segment. The central segment area is the leftover area after the rest of the segments have been calculated. The weighting factor for each segment is then calculated and applied to a model profile to mimic the laser moving across. The central segment (largest weighting factor) applies to the data point in the center, while the segments towards the outside apply to the data points on either side (smallest weighting factor). The central data point is then adjusted based on the averaging, leading to a smeared profile.

Supplementary note 2:

Calculating Re of the growth fluid

Density (kg/m^3)	587<br>calculated from<br>Burnham et al.[13]
Viscosity (Pa s)	6.75x10^{-5}<br>extrapolated from<br>Pioro & Mokry[14]
Pressure (MPa)	200
Temperature (°C)	600
k_{eq}	150
k_{eff}	8.75
L	0.0075

Supplementary Table 1. **Table of parameters used in the calculations.**

Supplementary Equations[15]:

$$Sh = 1 + \sqrt[3]{Re\ Sc + 1}\left(\frac{0.096\ \sqrt[3]{Re}}{\frac{7}{Re^2} + 1}\right) \qquad Supplementary\ Eq. 3$$

$$Sc = \frac{\eta}{\rho\ D} \qquad Supplementary\ Eq. 4$$

By rearranging Eq. 4 (main text) to solve for δ:

$$\delta = -\frac{D}{R}\ln\left(\frac{\frac{k}{k_{eff}} - k}{1 - k}\right) \qquad \textit{Supplementary Eq.} 5$$

We can calculate a boundary layer thickness. Plugging this value into the mathematical definition of the boundary layer thickness, δ = *L* / *Sh*, and using the relationship between *Sh* and *Re* shown above, one *Re* will correspond to one δ.

Supplementary references

1. Ayuso, R. A. *et al.* The role of ridge subduction in determining the geochemistry and Nd--Sr--Pb isotopic evolution of the Kodiak batholith in southern Alaska. *Tectonophysics* **464**, 137–163 (2009).
2. Greenfield, J. E., Musgrave, R. J., Bruce, M. C., Gilmore, P. J. & Mills, K. J. The Mount Wright Arc: a Cambrian subduction system developed on the continental margin of East Gondwana, Koonenberry Belt, eastern Australia. *Gondwana Res.* **19**, 650–669 (2011).
3. Wildner, W., Lima, E. F., Nardi, L. V. S. & Sommer, C. A. Volcanic cycles and setting in the Neoproterozoic III to Ordovician Camaquã Basin succession in southern Brazil: characteristics of post-collisional magmatism. *J. Volcanol. Geotherm. Res.* **118**, 261–283 (2002).

4. Survey, U. S. G. Geochemistry of rock samples from the National Geochemical Database. *US Geol. Surv. Open File Rep.* **97**, (2008).

5. Hartlaub, R. P., Heaman, L. M., Ashton, K. E. & Chacko, T. The Archean Murmac Bay Group: evidence for a giant archean rift in the Rae Province, Canada. *Precambrian Res.* **131**, 345–372 (2004).

6. Kamei, A. Geology and petrography of the Abukuma granites in the Hiyama district, Fukushima Prefecture, NE Japan. *Bull. Geol. Surv. Japan* **54**, 395–409 (2003).

7. Kamei, A. Geology and petrography of the Abukuma granites in the Funehiki area, Fukushima Prefecture, NE Japan. *J. Geol. Soc. Japan* **109**, 234–251 (2003).

8. Kirschbaum, A., Hongn, F. & Menegatti, N. The Cobres plutonic complex, eastern Puna (NW Argentina): petrological and structural constraints for lower Paleozoic magmatism. *J. South Am. Earth Sci.* **21**, 252–266 (2006).

9. Knox, K. L. The Never Summer igneous complex: Evolution of a shallow magmatic system. (University of Colorado at Boulder, 2006).

10. Maxeiner, R. O., Corrigan, D., Harper, C. T., MacDougall, D. G. & Ansdell, K. Paleoproterozoic arc and ophiolitic rocks on the northwest-margin of the Trans-Hudson Orogen, Saskatchewan, Canada: their contribution to a revised tectonic framework for the orogen. *Precambrian Res.* **136**, 67–106 (2005).

11. O'Leary, M. S., Lira, R., Dorais, M. J. & Tassinari, C. C. G. Post-collisional lamprophyric event in Sierra Norte, Córdoba, Argentina: Mineralogical, geochemical and isotopic characteristics. *J. South Am. Earth Sci.* **28**, 277–287 (2009).

12. Polin, V. F., Ras, V., Sakhno, G., Ekimova, N. I. & Sandimirova, G. P. Pantellerite-comendite-alkali granite association of the Paleogene bimodal series of the Okhotsk-

Chukotka volcanoplutonic belt. in *Doklady earth sciences* **407**, 412 (2006).

13. Burnham, C. W., Holloway, J. R. & Davis, N. F. The specific volume of water in the range 1000 to 8900 bars, 20 to 900 C. *Am. J. Sci.* **267**, 70–95 (1969).

14. Pioro, I. & Mokry, S. Thermophysical properties at critical and supercritical pressures. in *Heat Transfer-Theoretical Analysis, Experimental Investigations and Industrial Systems* (IntechOpen, 2011).

15. Zhang, Y. & Xu, Z. Kinetics of convective crystal dissolution and melting, with applications to methane hydrate dissolution and dissociation in seawater. *Earth Planet. Sci. Lett.* **213**, 133–148 (2003).
