# Supplementary material for: Episodes of fast crystal growth in pegmatites
Source: Nat Commun. 2020 Oct 5;11:4986. doi: 10.1038/s41467-020-18806-w (PMC7536386; doi:10.1038/s41467-020-18806-w)
Supplement: Supplementary file 3 — Description of Additional Supplementary Files [file 41467_2020_18806_MOESM3_ESM.pdf]

Description of Additional Supplementary Files

File Name: Supplementary Data 1
Description: Chemical analysis data
